# Identification of a Potent Endothelium-Derived Angiogenic Factor

**DOI:** 10.1371/journal.pone.0068575

**Published:** 2013-07-29

**Authors:** Vera Jankowski, Markus Tölle, Thi Nguyet Anh Tran, Markus van der Giet, Mirjam Schuchardt, Kerstin Lehmann, Doreen Janke, Burkhard Flick, Alberto Arduan Ortiz, Niño Maria Dolores Sanchez, Martin Tepel, Walter Zidek, Joachim Jankowski

**Affiliations:** 1 Charité-Universitaetsmedizin Berlin, Medizinische Klinik IV (CBF), Berlin, Germany; 2 Julius Wolff Institute and Berlin-Brandenburg Center for Regenerative Therapies, Charite – Universitaetsmedizin Berlin, Berlin, Germany; 3 Charité-Universitaetsmedizin Berlin, Institute of Toxicology (CBF), Berlin, Germany; 4 IIS-Fundacion Jimenez Diaz-UAM and IRSIN, Madrid, Spain; 5 IdiPAZ, Madrid, Spain; 6 University of Southern Denmark, Institute of Molecular Medicine, Odense, Denmark; UT-Southwestern Med Ctr, United States of America

## Abstract

The secretion of angiogenic factors by vascular endothelial cells is one of the key mechanisms of angiogenesis. Here we report on the isolation of a new potent angiogenic factor, diuridine tetraphosphate (Up_4_U) from the secretome of human endothelial cells. The angiogenic effect of the endothelial secretome was partially reduced after incubation with alkaline phosphatase and abolished in the presence of suramin. In one fraction, purified to homogeneity by reversed phase and affinity chromatography, Up_4_U was identified by MALDI-LIFT-fragment-mass-spectrometry, enzymatic cleavage analysis and retention-time comparison. Beside a strong angiogenic effect on the yolk sac membrane and the developing rat embryo itself, Up_4_U increased the proliferation rate of endothelial cells and, in the presence of PDGF, of vascular smooth muscle cells. Up_4_U stimulated the migration rate of endothelial cells via P2Y2-receptors, increased the ability of endothelial cells to form capillary-like tubes and acts as a potent inducer of sprouting angiogenesis originating from gel-embedded EC spheroids. Endothelial cells released Up_4_U after stimulation with shear stress. Mean total plasma Up_4_U concentrations of healthy subjects (N = 6) were sufficient to induce angiogenic and proliferative effects (1.34±0.26 nmol L^-1^). In conclusion, Up_4_U is a novel strong human endothelium-derived angiogenic factor.

## Introduction

Vasculature in adult mammals is mainly quiescent; however, new blood vessel formation is required for timely tissue repair and remodeling after injury [1]. The formation of new blood vessels is an essential process in the life of higher organisms. Development, reproduction, wound healing, communication of humoral signals, transport of nutrients and waste products all require angiogenesis [2]. The process of angiogenesis involves migration, proliferation, differentiation, and adhesion of multiple cell types, including endothelial, mural, and inflammatory cells [3], [4].

However, disease processes such as cancer growth [5], diabetic retinopathy or chronic inflammation are also dependent on angiogenesis [6]. Hence, the humoral mechanisms of angiogenesis have attracted increasing interest [7]. Among those, interest has focused on peptidic angiogenic factors such as the vascular endothelial growth factors, hepatocyte growth factor or fibroblast growth factor, and non-peptidic, low molecular angiogenic factors such as adenosine or hypoxic metabolites, e. g. lactate or pyruvate, which mediate hypoxia-induced angiogenesis. Although various cell types are required in the humoral regulation of angiogenesis; the contribution of vascular endothelial cells is probably the most important. However, our knowledge about the mediators secreted by endothelial cells inducing angiogenesis is just at the beginning. Unravelling these mediators involved in angiogenesis would offer therapeutic options to ameliorate disorders that are currently leading causes of mortality and morbidity, including cardiovascular diseases, cancer, chronic inflammatory disorders, diabetic retinopathy, excessive tissue defects, and chronic non-healing wounds. The knowledge of the endogenous mediators involved provides numerous opportunities for therapeutic intervention [8].

Therefore, we screened the secretome of human endothelial cell cultures for further, yet unknown angiogenic factors by using the culture of rat embryos including their yolk sac with its developing vascular system. The embryos were cultured during organogenesis, when angiogenesis is a fundamental process [9], [10]. The whole embryo culture (WEC) has been used before to study different growth factors, e.g. vascular endothelial growth [11], or to demonstrate the impact of different genes involved in angiogenesis [12]. We showed that incubation with alkaline phosphatase just partly reduced and blockade of purine P2 receptors markedly reduced the angiogenic effect of the endothelial secretome. Subsequently, diuridine tetraphosphate (Up_4_U) was identified as the responsible angiogenic factor.

## Materials and Methods

### Chemicals

HPLC water (gradient grade) and acetonitrile were purchased from Merck (Germany), all other substances from Sigma Aldrich (Germany).

### Culture of Endothelial Cells

Human endothelial cells from dermal microvessels (HMEC-1) present the first immortalized human microvascular endothelial cell line that retains the morphologic, phenotypic, and functional characteristics of normal human microvascular endothelial cells [13]. These cells were cultured in MCDB 131 medium supplemented with 100 U ml^-1^ penicillin/streptomycin, 1% (v/v) L-glutamine and 7.5% (v/v) fetal bovine serum. Experiments comparing the phenotypic characteristics of HMEC-1 cells with human dermal microvascular endothelial cells or human umbilical vein endothelial cells revealed that HMEC-1 cells show features of both, small- and large-vessel endothelial cells [13]. On day 0 cells were placed into 175 cm^2^ cell-culture flasks (Nunc Inc., Germany) and were stimulated on day 2 at approximately 70% confluency. Confluent cultures of HMEC-1 cells showed typical cobblestone appearance and were further characterized by the expression of Willebrand factor, endothelial nitric oxide synthase, VEGF receptor 1 (FLT-1) and absence of smooth muscle α-actin staining [14]. Primary human umbilical vein endothelial cells (HUVEC) were commercially obtained (Promocell, Germany) and expanded with endothelial growth medium (Promocell, Germany). Experiments were performed with cells grown for no more than four passages.

### Stimulation of Cultured Endothelial Cells

Cell-culture flasks of endothelial cells (n = 30) were washed three times with a physiological salt solution. After addition of 15 ml physiological salt solution, the cell-culture flasks of endothelial cells were exposed to shear stress for 10 min by using a horizontally shaking machine [15]. The supernatant was collected and pooled after shear stress stimulation. Aliquots of the resulting supernatants were incubated with immobilized alkaline phosphatase as described earlier [16]. The supernatant was deproteinized with perchloric acid (final concentration 0.6 mol L^-1^) and centrifuged (3,500 U min^-1^; 4°C; 5 min). Perchloric acid was precipitated by adding KOH (pH 9.5). The precipitated proteins and the insoluble reaction product KClO_4_ were removed by centrifugation (3,500 U min^-1^; 4°C; 5 min). Aliquots of the supernatant were neutralized before testing in the bioassay. For control reactions, 30 cell-culture flasks of endothelial cells were washed three times with 15 ml of a physiological salt solution by avoiding mechanical stress. Salt solution was added extremely slowly. After washing, 15 ml physiological salt solution was added to the endothelial cells. 10 min later, the supernatant was collected and pooled.

### Application of Shear Stress by Cone-and-plate Viscometer

Cultured human umbilical vein endothelial cells (HUVEC) were subjected to shear stress in a cone-and-plate viscometer [17], [18]. The secretome of HUVEC exposed to 1 dyn cm^-2^ (low, 0.1 N m^-2^) and 30 dyn cm^-2^ (high, 3.0 N m^-2^) shear stress for 24 h were compared to the secretome of static control cells (0 dyn cm^-2^). Cell culture medium supplemented with 5% dextran T-70 (Sigma-Aldrich, Germany) was added to the cell culture medium 1 h prior to biomechanical stimulation to increase the viscosity 2.95-fold to 0.02065 dyn s^-1^ cm^-2^. Dextran had no influence on the expression of genes studied.

### Whole Embryo Culture (WEC)

This study was approved by the “*Ethical Committee Charité*”. All animal procedures conducted were in accordance with the guideline for the care and use of laboratory animals by the “*Research Institute of Experimental Medicine*” (FEM) of the Charité (Germany), approved by the “*Ethical Committee Charité*”. Wistar rats unilever (Bor:isw/SPF, TNO; Harlan-Winkelmann, Germany) were kept under specific pathogen-free conditions at a constant day and night cycle of 12 hours starting at 9∶00 a.m. and 9.00 p.m and the following 24 h were designated as day 0 of pregnancy when sperm was detected in the vaginal smear.

On gestational day 9.5, the gravid rats were sacrificed by decapitation and the rat embryos were prepared and cultured according to a method previously published in detail [19]. The preparation of the embryos was performed in HBSS, they were placed in groups of four into sealed culture flasks (50 ml) containing 7 ml of the culture medium. The culture medium consists of 15% HBSS and 85% donor bovine serum (Quad Five, USA), supplemented with 1.57 mg ml^-1^ D-glucose (Merck Eurolab, Germany) and 75 µg ml^-1^ L-methionine (Sigma-Aldrich, Germany). For incubation the culture flasks were placed for 48 h into a roller device (Memmert, Germany) at a speed of 25 rpm and a temperature of 38.5°C. Initiating the culture, the flasks were gassed with 10% O_2_, 5% CO_2_, and 85% N_2_. After 36 h the oxygen concentration was raised to 50%.

After 48 h of culture the embryos were evaluated for their growth (crown-rump length and protein content) and their differentiation (number of somites and morphological score) [19] using a dissection microscope. Finally the development of the yolk sac was estimated with special attention to its blood vessels system. After the morphological evaluation of the cultured embryos and their corresponding yolk sacs these tissues have been frozen and immediately stored at−80°C.

### Chromatographic Analysis of the Supernatants of Endothelial Cells

Supernatants of stimulated endothelial cells were fractionated by a series of reversed-phase and affinity chromatographic steps. Triethylammonium acetate (40 mmol L^-1^ final concentration) was added to the supernatants. pH was titrated to 6.5. Next, two C18 reversed-phase columns (Chromolith Performance, RP C18e, 100×4.6 mm, Merck, Germany) connected in series were used to concentrate the supernatant of stimulated and unstimulated endothelial cells. Non-binding substances were removed with triethylammonium acetate. Binding substances were eluted stepwise with 25% acetonitrile (ACN), in water at a flow rate of 1.0 ml min^-1^. Unless specified the chromatographic eluent was monitored at 254 nm using a (make and model of detector). The eluate was retained and frozen at –80°C and lyophilised.

The eluate of the preparative reversed-phase chromatography column was purified further with affinity chromatography. The affinity chromatography gel, phenyl boronic acid coupled to a cation exchange resin (Biorex 70, Bio-Rad, USA), was synthesized according to Barnes et al.[20]. The affinity resin was packed into a glass column and equilibrated with 0.3 mol L^-1^ ammonium acetate (pH 9.5). The pH of the eluate from the preparative reversed-phase chromatography was adjusted to pH 9.5 and loaded to the affinity column. The column was washed with an ammonium acetate solution with a flow rate of 1.0 ml min^-1^. Binding substances were eluted with 1 mmol L^-1^ HCl solution. The eluate was retained and frozen at −20°C.

1 mol L^-1^ triethylammonium acetate was added to the eluate of the affinity chromatography (final concentration: 40 mmol L^-1^). The eluate of the affinity chromatography was injected into a reversed phase high performance liquid chromatography (Chromolith RP-18e 100-4.6, Merck, Germany) for desalting. After removal of substances not binding to the column with aqueous 40 mmol L^-1^ triethylammonium acetate, the absorbed substances were eluted with 20% acetonitrile (ACN) in water at a flow rate of 1.0 ml min^−1^. Each eluate was frozen at –80°C and lyophilized.

The lyophilised eluate was then dissolved in 40 mmol L^-1^ triethylammonium acetate (eluent A) and injected in two reversed phase columns (Chromolith RP-18e 100-4.6 Merck, Germany) connected in series. 80% acetonitrile (eluent B) and the following gradient were used for the elution: 0–10% B 40 min, 10–100% B 1 min, 100% B 2 min. The flow rate was 1.0 ml min^-1^ and 1 ml fractions were collected.

### Determination of Recovery Rates

To calculate the recovery rate for Up_4_U, in a control experiment, either culture medium or plasma (40 ml) was spiked with Up_4_U (5 µg). These samples were fractionated as described above.

### Matrix Assisted Laser Desorption/Ionisation Mass Spectrometry (Maldi-MS)

The lyophilised fractions of the reverse-phase chromatography were analysed by matrix-assisted laser desorption/ionisation mass spectrometry (MALDI-MS) and MALDI fragment ion analysis using a Bruker Ultraflex TOF/TOF instrument (Bruker-Daltonics, Germany). The concentrations of the analysed substances were 1–10 µmol L^–1^ in double distilled water. 1 µl of the analyte solution was mixed with 1 µl of matrix solution (50 mg ml^–1^ 3-hydroxy-picolinic acid in water). Cation exchange beads (AG 50 W-X12, 200–400 mesh, Bio-Rad, Germany) were added to this mixture and equilibrated with NH_4_^+^ as a counter-ion to remove Na^+^ and K^+^ ions. 1 µl of each fraction was prepared on a prestructured MALDI sample support (MTP AnchorChip™ 400/384, Bruker-Daltonics, Germany) [21] and dried gently on an inert metal surface before introduction into the mass spectrometer.

Mass-spectrometric measurements were performed on a Bruker Ultraflex-III TOF/TOF instrument (Bruker-Daltonics, Germany). The instrument was equipped with a Smart beam™ laser operating with a repetition-rate of 100–200 Hz. On average, the presented spectra are the sums of 300 single-shot spectra for MS mode, and 1,000 for MS/MS mode. Argon was used as collision-induced dissociation (CID) gas. Mass spectra of positively charged ions were analysed in the reflector mode using delayed ion extraction. Fragment ion spectra were recorded using the LIFT option of the instrument. The calibration constants were determined using standard peptides prepared on positions adjacent to the sample, resulting in an error of <50 ppm for the recorded mass spectra. The dinucleoside polyphosphate ApcpcpA was added to the sample as internal standard in the case of kinetic measurements by using MALDI mass spectrometry. Local differences in the Up_4_U-concentration on the MALDI spot were thereby eliminated [22].

### Enzymatic Cleavage Experiments

Enzymatic cleavage experiments were performed as described elsewhere [16], [23]. Briefly, 5-nucleotide hydrolase (3 mU) from Crotalus durissus (Sigma-Aldrich, Germany), 3`-nucleotide hydrolase (1 mU) from calf spleen (Sigma-Aldrich, Germany) and alkaline phosphatase (1 mU) from calf intestinal mucosa (Fluka, Germany), respectively were mixed with 50 µl NaHCO_3_ and activated CNBr-Sepharose 6 MB beads (Amersham-Pharmacia Biotech, Sweden). The mixture was incubated for 2 hours at room temperature. After incubation, the beads were washed 3 times with double distilled water. Aliquots of the fractions from the reversed phase chromatography were incubated with these enzyme-beads for 2 hours at room temperature. Aliquots of the reaction mixture were examined by MALDI-MS. 40–50 single spectra were accumulated to improve the signal-to-noise ratio [23]. Sample preparation and measurements were done at the same conditions as for the original samples.

### Synthesis of Diuridine (5′, 5′) Tetraphosphate

Up_4_U was synthesized according to Ng and Orgel [24]. Uridine 5′-diphosphate (UDP; 50 mmol L^-1^), (N-[2-hydroxyethyl]-piperazine-Ń-[2-ethanesulfonic acid]) (HEPES; 2 mol L^-1^, 1-ethyl-3-(3-dimethylamino-propyl)carbodiimide (2.5 mol L^-1^) and magnesium chloride (MgCl_2_; 125 mmol L^-1^) were dissolved in water, thoroughly mixed with a vortex mixer and incubated at 37°C at pH 6.5 for 48 h. Purification of chemically synthesized dinucleoside (5′,5′) polyphosphates was performed as described elsewhere [25]. Briefly, the synthesized dinucleoside polyphosphates were concentrated on a C18 reversed phase column (LiChroprep, 310×25 mm, 65-40 µm, Merck, Darmstadt, Germany) using 40 mmol L^-1^ aqueous triethylammonium acetate (TEAA) in water (eluent A; flow rate: 2.5 ml min^-1^). After removing non-binding substances with eluent A (flow rate: 2.5 ml min^-1^), nucleotides were eluted with 36% acetonitrile in water (eluent B; flow rate: 2 ml min^-1^). The eluate was lyophilized and stored frozen at−80°C. The lyophilized eluate of the preparative reversed phase chromatography was dissolved in aqueous 40 mmol L^-1^ triethylammonium acetate solution and injected on two C18 reversed phase columns connected in series (Supersphere, 300×8 mm, 4 µm, Merck, Germany) which were equilibrated with aqueous 40 mmol L^-1^ triethylammonium acetate (carrier). The carrier was pumped through the system with a flow rate of 100 µl min^-1^ during injection of the sample. After the injection was finished, n-butanol (160 mmol L^-1^) in 40 mmol L^-1^ triethylammonium acetate was used as displacer (flow rate: 200 µl min^-1^). The fraction size was 1.9 ml. Each fraction of the displacement-chromatography possibly containing dinucleoside polyphosphates was lyophilized, dissolved in 1 ml 20 mmol L^-1^ K_2_HPO_4_ in water, pH 8, (eluent A) and chromatographed by using an anion-exchanger (column: UNO Q-12, BioRad, Germany)(eluent B: 20 mmol L^-1^ K_2_HPO_4_ and 1 mol L^-1^ salt (pH 8) in water; gradient: 0–10 min: 0–5% B; 10–10 min: 5–35% B; 100–101 min: 35–100% B; flow rate: 3.0 ml min^-1^; UV absorption wavelength: 254 nm). The fractions of the anion-exchange chromatography were desalted by HPLC reversed phase C18 chromatography. The reversed phase column (Chromolith™ Performance RP-18e100-4.6, Merck, Germany) was equilibrated with eluent A (40 mmol L^-1^ triethylammonium acetate). Each sample dissolved in 40 mmol L^-1^ triethylammonium acetate was pumped with a flow rate of 1.0 ml min^-1^ onto the column. After 20 minutes washing the column with 30 ml eluent A, the substances were eluted with 32% acetonitrile in water (eluent B). The resulting fractions were lyophilized and stored at −80°C. The lyophilized fractions from the HPLC reversed phase C18 chromatography were examined by MALDI-MS.

### Isolation and Identification of Diuridine Tetraphosphate in Human Plasma

The blood collection was approved by the ethical committee of the Charité. The probands gave their written consent. Peripheral blood (20 ml) was drawn from the cubital vein in six healthy subjects and was collected in tubes containing K_2_-EDTA (7.2 mg). The mean age of the subjects (m/f: 3/3) was 31.8±2.8, systolic blood pressure 118±2 (mmHg), diastolic blood pressure 73±3 (mmHg)(each mean ± SEM). The blood samples were centrifuged at 2,100 g for 10 min at 4°C for isolation of plasma, after a standardized interval of 15 min post sampling. 5 µg of a diinosine tetraphosphate (Ip_4_I) was added as internal standard and used to compensate for any losses during purification. The plasma was deproteinized with 0.6 mol L^-1^ (final concentration) perchloric acid and centrifuged (2,100 g, 4°C, 5 min). After adjusting pH to 7.0 with 5 mol L^-1^ KOH the precipitated proteins and KClO_4_ were removed by centrifugation (2,100 g, 4°C, 5 min).

### Isolation and Identification of Diuridine Tetraphosphate from Human Plasma

Triethylammonium acetate (TEAA) in water was added to the deproteinized plasma to a final concentration of 40 mmol L^-1^. This mixture was fractionated to homogeneity by reversed phase chromatographic and affinity chromatographic methods comparable to the methods used for the chromatographic analysis of the endothelial secretome. Up_4_U was identified on the basis of its retention time as compared to synthetic Up_4_U. The lyophilised fractions from the reverse phase HPLC with TEAA as the ion-pair reagent were further separated by analytic reverse phase HPLC using tetrabutylammonium hydrogensulfate (TBA) as the ion-pair reagent. The fractions, dissolved in 150 µl of 2 mmol L^-1^ TBA and 10 mmol L^-1^ K_2_HPO_4_ (pH 6.5), were injected into a reverse phase HPLC column (Chromolith^TM^Performance, RP-18e; 100-4.6 mm; Merck, Germany). Acetonitrile (80% (v/v) in water: eluent B) and the following gradient was used for the elution: 0–41 min: 0–30% eluent B; 41–41.5 min: 30–100% eluent B; 41.5–44.5 min: 100% eluent B; flow: 3 ml min^-1^. The concentrations of Up_4_U were calculated using calibration curves created with synthetic Up_4_U.

### Detection of Endothelial Cell Proliferation

To detect cell proliferation after treatment with Up_4_U, HUVECs were incubated in the presence of 10 µmol L^-1^ of the thymidine analogue 5′bromo-2′deoxyuridine, following the manufacturer’s protocol (BrdU, Roche, Germany). Briefly, HUVEC were seeded into 96 well plates at a cell density of 2,000 cells well^-1^. Cells were treated with increasing concentrations of Up_4_U (0, 0.1, 1, 10, 100 nmol L^-1^) in endothelial cell growth medium containing 0.1% FBS for 24 h. To inhibit the Up_4_U effect, cells were treated with 100 µmol L^-1^ suramin (Sigma-Aldrich, Germany) in the presence of Up_4_U. Cells were labelled with 10 µmol L^-1^ BrdU in the last 4 h of treatment. After fixation, cells were incubated with Blocking Reagent (Roche, Germany) for 30 min to reduce unspecific binding of the antibody conjugate. Incorporated BrdU was detected with monoclonal anti-BrdU-POD antibody (30 min at RT) and ABST substrate (15–30 min at RT). Absorbance was measured at 370 nm (reference 492 nm). The experiment was carried out with 6 wells for each treatment and was repeated three times.

### Migration Assays

Migration assays were performed using a disposable 96-well ChemoTX chamber (Neuro Probe, USA) with 8 µm pores. Prior to each experiment filters were coated with 0.1 mg ml^-1^ collagen type I and placed at 37°C for 1 h to polymerize. For each experiment quiescent cells were loaded with 1 µmol L^-1^ Calcein-AM (Invitrogen, USA) in DMEM containing 0% FCS and 1.25 mmol L^-1^ probenecid for 1 h to enable a fluorescent detection of the cells. After loading with the dye cells were harvested with trypsin and resuspended in DMEM containing 0% FCS and 1.25 mmol L^-1^ Probenecid. The lower wells of the plate were filled with test substances, covered with the filter and 2.5×10^4^ cells were placed on the filter sites. The chamber was incubated for 5 h at 37°C and 5% CO_2_. Following incubation, non-migrated cells were mechanically removed and the filter was measured using the fluorescence signal of calcein at 485 nm (excitation) and 535 nm (emission) in a fluorescence plate reader (Mithras LB 940, Berthold Technologies, Germany).

### Detection of Vascular Smooth Muscle Cell Proliferation

Proliferation was determined by bromodeoxyuridine (BrdU) incorporation during DNA synthesis, using a BrdU-ELISA (Roche Diagnostics, Switzerland). Cells were plated at a density of 2,500 cells well^-1^ in 96-well plates and cultured for 24 h. Following 24 h in serum-reduced medium (0.5% FCS), the culture medium was removed and fresh medium containing the test substances was added to the growth-arrested cells for another 24 h. BrdU was offered in the last 4 h of the incubation time. After stimulation cells were fixed, incubated with anti-BrdU-POD antibody and washed according to manufacturer`s instructions. Substrate solution was added to the wells and the luminescence signal representing the BrdU incorporation was recorded immediately by a luminescence plate reader (Mithras LB 940, Berthold Technologies, Germany).

### Tube Formation

The tube formation assay was carried out with the µ-slide angiogenesis system from Ibidi (Integrated BioDiagnostics, Germany). The µ-slides were coated with growth-factor reduced BD Matrigel (BD Biosciences, Japan) and placed at 37°C for 1 h to polymerize. HMECs were harvested and resuspended in growth factor free MCDB 131 medium at a density of 2×10^5^ cells ml^-1^. From this solution 50 µl were applied in each µl-slide well and incubated for 6 h. Tube formation was measured using microscopic images of five different areas. Tubular length and total number of tubes were quantified.

### Sheroid Sprouting Assay

HUVEC cells were cultured in endothelial cell culture medium consisting of endothelial basal cell growth medium containing (ECM2), 2% FBS and endothelial cell growth supplements. The cells were cultured to 90% confluency at 37°C and 5% CO_2_ and used from passage 2 to passage 4. Endothelial spheroids were generated as described previously [26]. Briefly, human umbilical vein endothelial cells (2,500–3,000 cells per spheroid) were resuspended in endothelial cell culture medium containing 20% carboxymethylcellulose and plated in nonadherent round-bottom 96-well plates for 24 hours allowing single spheroid aggregation. Spheroids were harvested and combined in a 1.5 ml Eppendorf tube. Cell culture supernatant was removed after centrifugation for 1 min at 500 x g. 30 spheroids were embedded into 120 µl collagen gels in 24-well plates [27]. For collagen stock solution, 8 vol rat tail collagen type I (Collaborative Medical Products, US) was mixed with 1 vol. 10x PBS (Sigma-Aldrich, Germany) and 1 vol. 0.1 N NaOH to adjust to pH 7.4 at room temperature. This stock solution was then mixed with an equal volume of endothelial basal growth medium (ECM2, Lonza, Germany) containing 40% FBS (Lonza, Germany) and 0.5% carboxymethylcellulose to prevent spheroid sedimentation during collagen gel polymerization. Spheroid containing gels were allowed to polymerize for 20 min at 37°C and 5% CO_2_ and then overlaid with endothelial cell culture medium (ECM), supplemented with Up4U concentrations as indicated or 20 ng ml^-1^ VEGF (Sigma-Aldrich, Germany). After 24 hours in vitro sprout formation was evaluated in phase-contrast images (Leica, Germany) and quantified by SCORE image analysis (SCO Life Science, Germany).

### Phosphoprotein Detection for Map-Kinases

Serum-starved VSMCs were stimulated with Up_4_U for the indicated time points. After harvesting cells with ice-cold cell lysis buffer (Biorad, Munich, Germany), centrifuged for 20 min at 4°C and 13.000 rpm, supernatant was spiked with equal amount of assay buffer (Biorad, Munich, Germany). Protein amount of the lysates was determined with BCA™ assay kit (Pierce, Rockford, USA). Determination of phosphorylated as well as total protein was assayed using Luminex™ technology with the phospho-protein detection assay (Biorad, Munich, Germany).

### In-Vivo/Ex-Vivo Assay of Up4U Production in Isolated Aortic Rings

Thoracic and abdominal aorta was isolated from Wistar rats (n = 4). The surrounding fat tissue was removed and aortas were serially cross-sectioned into 1–2 mm rings. A total of 10–15 aortic rings were seeded into a 12-well plate and serum-starved in Opti-MEM for 24h to equilibrate their growth factor responses. Then the conditioned medium was collected (base-line) and fresh serum-free medium was added in the absence (Control) or presence of calcium ionophore (10 µmol L^-1^, Sigma) or endothelin 1 (0.1 nmol L^-1^, Sigma). Conditioned media was collected after 45 minutes, deproteinated and frozen until assayed.

### Statistical Methods

Data are given as mean values with standard error mean (SEM). All statistical analyses were done using SPSS software (Microsoft SPSS for Windows, version 12.0). The Wilcoxon-Mann-Whitney test was used for non-parametric statistical tests. p<0.05 (two-sided) was considered to indicate statistical significance.

## Results

**Figure 1:**
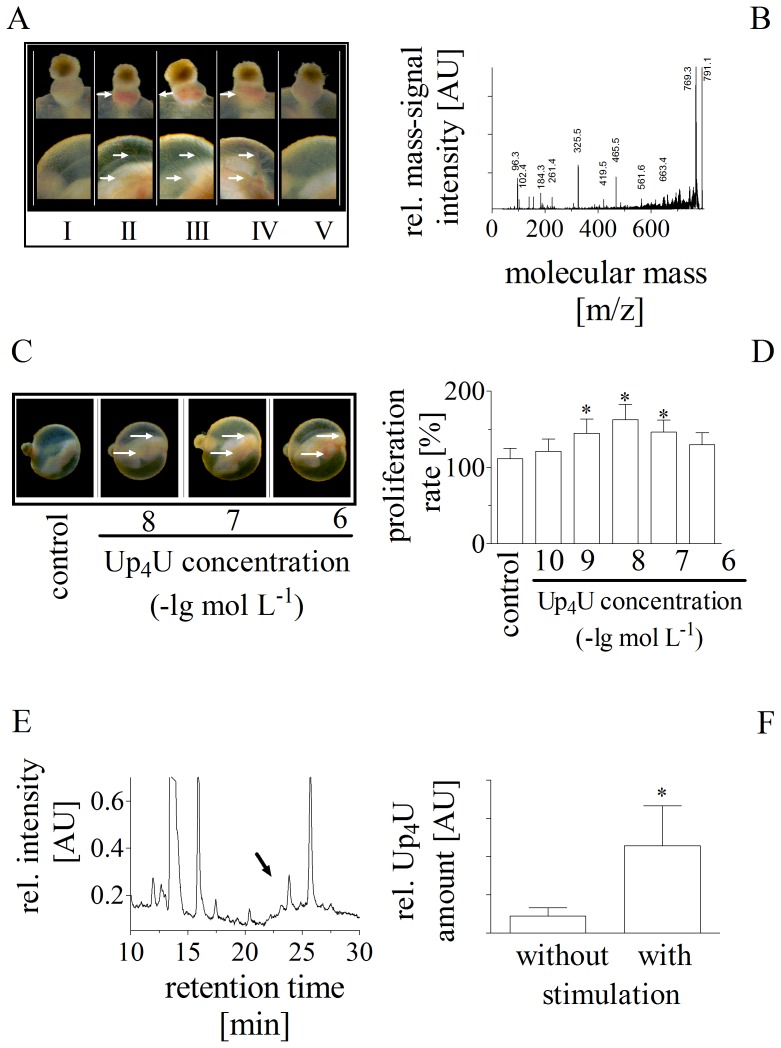
(**A**) Angiogenic effects of endothelial secretome in the rat embryo chorioallantoic membrane. The primitive placenta and yolk sac of rat embryos cultured during organogenesis under negative control conditions (HBSS and bovine serum) (I). The corresponding vascular system is underdeveloped and could be improved by angiogenic factors like VEGF as positive control (II). More complex and structured blood vessels and red staining caused by red blood cells in the blood vessels (marked by arrows). Morphologic evaluation of angiogenic effect of the endothelial secretome (III), of the endothelial secretome after incubation with alkaline phosphate (IV), and of the endothelial secretome after incubation with alkaline phosphate in the presence of suramin (V). (**B**) MALDI-TOF-TOF mass spectrum of the fraction from the analytical reversed-phase chromatography. (**C**) Enhanced vascularisation of rat embryonic yolk sac membranes induced by increasing Up_4_U concentrations after 48 h of culture. Typical result out of 5 similar experiments. (D) Effect of increasing Up_4_U concentration on proliferation rate of human endothelial cells (n = 7). (**E**) Reversed phase chromatography of the fraction of human plasma containing the remaining nucleotides after exclusion of mononucleotides. (**F**) Up_4_U release of cultivated endothelial cells after stimulation by a cone-and-plate viscometer with shear stress of 3 N m^-2^ (n = 11).

The screening approach using the chorioallantoic membrane of the developing rat embryo [11], [28] showed that the supernatant obtained from HMEC-1 stimulated by shear stress elicited an angiogenic effect in comparison to control (**Figure 1** **.A**). Gestational day 9.5 rat embryos were cultured for 48 h. This time period during embryogenesis covers a major part of organogenesis, when a complex vasculature is developed in the yolk sac as well as in the embryo itself. The most prominent blood vessels are located in the yolk sac surrounding the embryo. In the negative control cultivated with HBSS and bovine serum (**Figure 1** **.A.I**), the yolk sac exhibited an immature vascular network consisting of irregularly organised small vessels. The corresponding vascular system is developed by angiogenic factors like VEGF as positive control (**Figure 1** **.A.II**). By contrast, the primitive placenta and yolk sac of the embryos exposed to the secretome of endothelial cells showed a highly organized vasculature containing large and small vessels (**Figure 1** **.A.III**). Furthermore, the supernatant from endothelial cells were treated with immobilised alkaline phosphatase, which metabolises mono- but not dinucleoside polyphosphates. Incubation with the immobilised alkaline phosphatase had a slightly diminished effect on angiogenesis (**Figure 1** **.A.IV**). This effect was abolished in the presence of the unspecific P_2_-receptor antagonist suramin (**Figure 1** **.A.V**).

These experiments helped to choose the additional purification steps applied to endothelial cell supernatants. First, we deproteinized supernatants from stimulated endothelial cells to isolate fractions most likely containing endothelial-derived nucleotides. After deproteination, we desalted the supernatants by using a preparative reversed-phase chromatography chromatography (**Figure S1.A.)**. The 30% acetonitrile eluates of the reversed phase chromatography were fractionated by using a phenylboronate affinity column in order to separate mononucleotides from nucleotides containing at least two pairs of neighbouring cis-diol groups (**Figure S1.B.**). Afterwards, we fractionated the nucleotides containing at least two pairs of neighbouring cis-diol groups by analytical reversed-phase chromatography. The resulting chromatogram showed a single sharp UV peak **(Figure S1.C.**).

The MALDI-TOF-TOF mass spectrum obtained from the underlying fraction revealed a molecular mass of 791.4 Da (M+H^+^). **Figure 1** **.B** demonstrates the MALDI-TOF-TOF-MS/MS-LIFT-fragmentation mass-spectrum of the underlying substance. Each mass-fragments signal was attributable to a fragment of Up_4_U by using an in-house database and was identical with the MS/MS fragmentation mass-spectrum of synthetic Up_4_U as shown in **Table 1**, suggesting that Up_4_U was the substance under investigation. Molecular structure of Up_4_U is given in **Figure S1.D.**

**Table 1:** Molecular masses of Up_4_U fragments obtained by MALDI-TOF-TOF mass spectrometry (**Figure 1** **.B)**.

Fragment ions[M+H]^+^	Up_4_U isolated fromendothelial secretome(measured)	Up_4_U isolated fromplasma (measured)	Up_4_U fragmentmass (calculated)	synthetic Up_4_U(measured)
Ú-NH	96.3		96.0	96.0
C_4_H_6_O_3_	102.4	101.8	102.0	102.1
Ú+CHO	140.4	140.7	140.0	140.0
C_5_H_12_O_5_P	184.3	184.6	183.0	184.1
U	245.3	243.7	243.1	244.0
U+H_2_O	261.4	261.6	261.1	261.4
U-2 H_2_O	207.3	207.4	207.1	207.0
Up_1_	325.5	324.3	323.0	325.1
Up_2_-H_2_O	384.3	385.9	384.9	385.0
Up_2_	402.9	403.1	402.9	403.1
Up_2_+H_2_O	419.5	419.1	420.9	421.1
Up_3_-H_2_O	465.5	465.9	464.9	467.0
Up_3_	483.5	484.0	482.9	484.0
Up_4_	561.6	567.3	562.9	563.0
M- Up_4_	229.3	228.4	227.1	227.0
M-Ú-H_2_O	663.4	660.9	660.9	661.2
M–H_2_O	773.1	773.3	772.3	773.5
M-H_2_O–2 H	769.3	769.4	769.9	768.5
M	791.1	791.0	791.3	791.4

The first column shows the fragment masses measured by MALDI-TOF-TOF mass spectrometry; second column shows the fragments mass of Up_4_U isolated from the endothelial secretome; the third column the fragments mass of Up_4_U isolated from plasma; the fourth column shows the fragment masses calculated from their respective structures; the fifth column shows the fragments masses of synthesised Up_4_U. M^+^ = protonated parent ion; Ú =  uracil; U = uridine; p = phosphate group, e.g. Up_3_ =  UTP; w/o = without.

After identification and synthesis of Up_4_U, the angiogenic effects of synthetic Up_4_U were verified using the assay whole embryo culture system [11], [28]. **Figure 1** **.C** demonstrates the impact of Up_4_U on the morphological pattern of the vascularisation of the yolk sac membrane. In the negative control, the yolk sac exhibited an immature vascular network consisting of irregular organised small vessels. By contrast, the yolk sac of the embryos exposed to the Up_4_U showed a highly organized vasculature containing large and small vessels (**Figure 1** **.C**).

Since proliferation of endothelial cells is essential for angiogenesis, next, the effect of Up_4_U on the proliferation rate of endothelial cells was analysed. Up_4_U induced a strong concentration-dependent stimulation of the proliferation of human endothelial cells at low concentrations; the proliferative effect of Up_4_U is inhibited by a feedback mechanism at concentrations above 10^−7^ mol L^-1^ (**Figure 1** **.D**). The threshold effect of Up_4_U was obtained at a concentration of 1 nmol L^-1^.

To investigate whether Up_4_U plasma concentrations are sufficient to induced angiogenesis, we quantified Up_4_U plasma concentration in healthy subjects by reversed-phase chromatography (**Figure 1** **.E**). The mean age of the subjects (m/f: 3/3) was 31.8±2.8, systolic blood pressure 118±2 (mmHg), diastolic blood pressure 73±3 (mmHg)(each mean ± SEM). The mean (± SEM) peripheral venous plasma Up_4_U concentration was 1.34±0.26 nmol L^-1^ (N = 6).

Afterwards, we studied endothelial Up_4_U release under physiologic conditions using a cone-and-plate viscometer. Shear stress of 3 N m^-2^ for 24 h caused a strong increase in Up_4_U concentration in the endothelial secretome compared to control situation without shear stress application (**Figure 1** **.F**).

**Figure 2:**
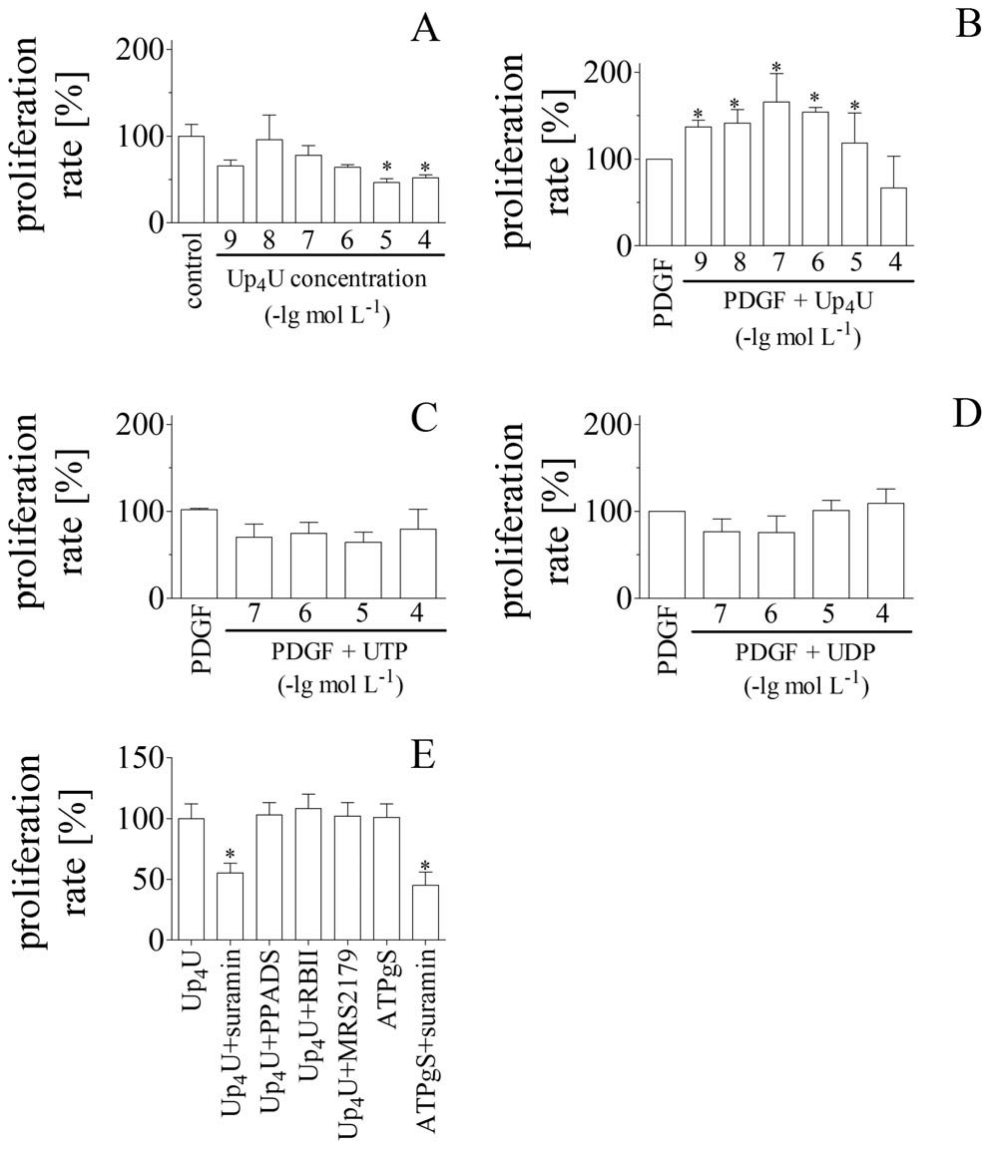
(**A**)Effect of increasing Up_4_U concentrations on proliferation rate of vascular smooth muscle cells in the absence of PDGF (n = 6). (**B**)Effect of increasing Up_4_U concentrations on proliferation rate of vascular smooth muscle cells in the presence of PDGF (10^−6^ mol L^-1^ PDGF each; n = 3). (**C**)Effect of increasing UTP concentrations on proliferation rate of vascular smooth muscle cells in the presence of PDGF (10^−6^ mol L^-1^ PDGF each; n = 3). (**D**)Effect of increasing UDP concentrations on proliferation rate of vascular smooth muscle cells in the presence of PDGF (10^−6^ mol L^-1^ PDGF each; n = 3). (**E**)Effect of Up_4_U (10^−7^ mol L^-1^) or ATPγS (10^−7^ mol L^-1^) in the presence of PDGF (10^−6^ mol L^-1^ PDGF each; n = 3) and suramin (10^−4^ mol L^-1^), PPADS (10^−5^ mol L^-1^), MRS2179 (10^−5^ mol L^-1^) or RBII (10^−5^ mol L^-1^) on proliferation rate of vascular smooth muscle cells.

To investigate whether Up_4_U not only increases the proliferation rate of endothelial cells, but also effects growth of vascular smooth muscle cells (VSMC), next the effect of Up_4_U on the VSMC proliferation rate was tested in the presence and absence of platelet-derived growth factor (PDGF). While Up_4_U had no direct effect on the VSMC proliferation rate at concentration below 10 µmol L^-1^ (**Figure 2** **.A**), Up_4_U strongly increased VSMC proliferation rate in the presence of PDGF at low concentration range (**Figure 2** **.B).** Up_4_U, but not its metabolites, UTP and UDP, caused this increasing effect, since UTP (**Figure 2** **.C**) and UDP (**Figure 2** **.D**) had no effect on the VSMC proliferation rate in the presence of PDGF. In the next step, we investigated potential receptors involved. Suramin significantly inhibited Up_4_U induced proliferation whereas PPADS, RBII and MRS2179 had no effect. From the inhibitory pattern of these non-selective P2Y-receptor antagonists, we had the idea of the P2Y2 receptor activated by Up_4_U. The non-hydrolizable ATP-γS is a selective agonist at the P2Y2 receptor. ATPγS is also able to induce a potent proliferation which is in part inhibitable by suramin (**Figure 2E**).

**Figure 3:**
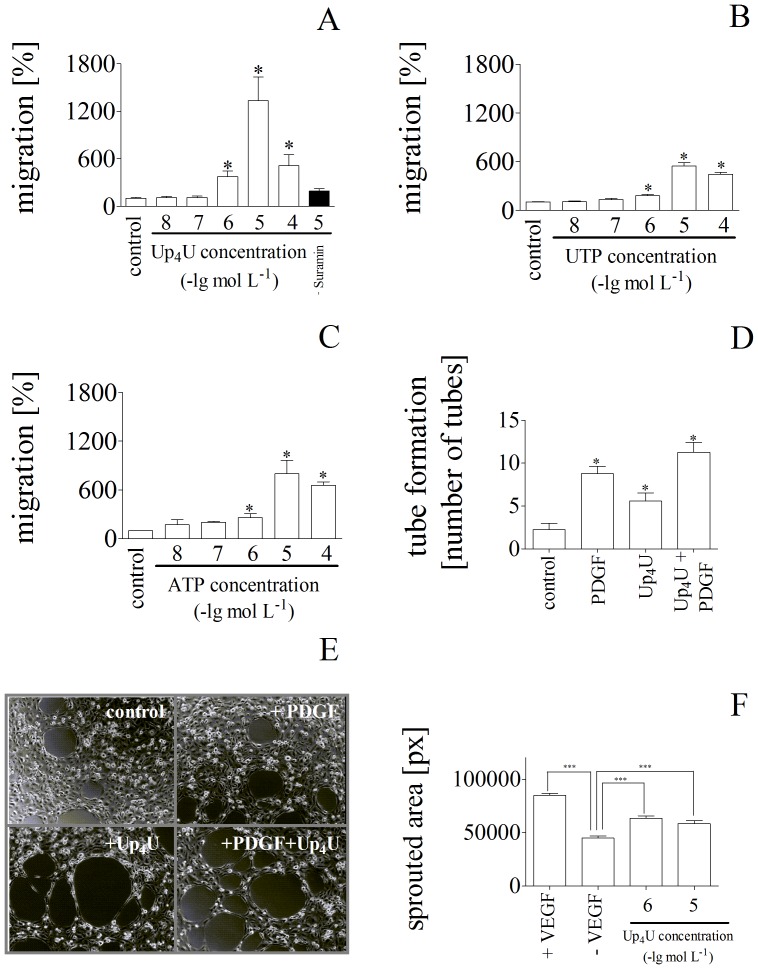
(**A**)Effect of increasing Up_4_U concentrations on migration rate of endothelial cells in the absence (open bar) and presence (filled bar) of suramin (n = 6). (**B**)Effect of increasing UTP concentrations on migration rate of endothelial cells (n = 3). (**C**)Effect of increasing ATP concentrations on migration rate of endothelial cells (n = 3). (**D**) Effect of increasing Up_4_U concentrations on tube-formation rate of endothelial cells (5 10^−5^ mol L^-1^ Up_4_U and 10^−7^ mol L^-1^ PDGF as indicated in the figure; n = 12). (**E**)Effect of Up_4_U on phenotype of endothelial cells. Representative microscopic images of endothelial cell were exposed to (a) control conditions, (b) PDGF, (c) Up_4_U, and (d) PDGF and Up_4_U for 6 h incubation time (5 10^−5^ mol L^-1^ Up_4_U and 10^−7^ mol L^-1^ PDGF (10ng ml^-1^)). (**F**)Quantification of 3-dimensional *in vitro* angiogenesis with collagen gel-embedded spheroids of EC. Spheroids were embedded into collagen gels with Up_4_U and with or without VEGF. The cumulative length of all the sprouts originating from an individual spheroid was quantified after 24 h by semiautomatic image analysis.

Since migration of endothelial cells is essential for neovascularization as well as proliferation, the effect of Up_4_U on migration rate was then analysed. Up_4_U induced a concentration-dependent increase in the migration rate of endothelial cells (open bars of **Figure 3** **.A**), which was abolished in the presence of suramin, indicating the involvement of the P2Y2-receptors in this effect (filled bar of **Figure 3A**). The Up_4_U effect on migration rate was stronger than the effects of UTP (**Figure 3** **.B**) and ATP (**Figure 3** **.C**).

To investigate whether Up_4_U affects the ability of endothelial cells to form capillary-like tubes, endothelial cells were exposed to Up_4_U for 6 h and examined for tube formation microscopically. Up_4_U produced an increased number of tubes compared to control (**Figure 3** **.D**). The tube formation by Up_4_U was additive to that of PDGF (**Figure 3** **.D**). Characteristic microscopic images are given in **Figure 3** **.E**.

To further analyze the effects of Up_4_U on endothelial functions and responsiveness, we performed experiments in gel angiogenesis with EC spheroids. Spheroids were embedded in collagen gels and stimulated with Up_4_U or VEGF as positive control. The cumulative length of outgrowing capillary-like sprouts was quantified after 24 h. Up_4_U acts as a potent inducer of sprouting angiogenesis originating from gel-embedded EC spheroids (**Figure 3** **.F**).

**Figure 4:**
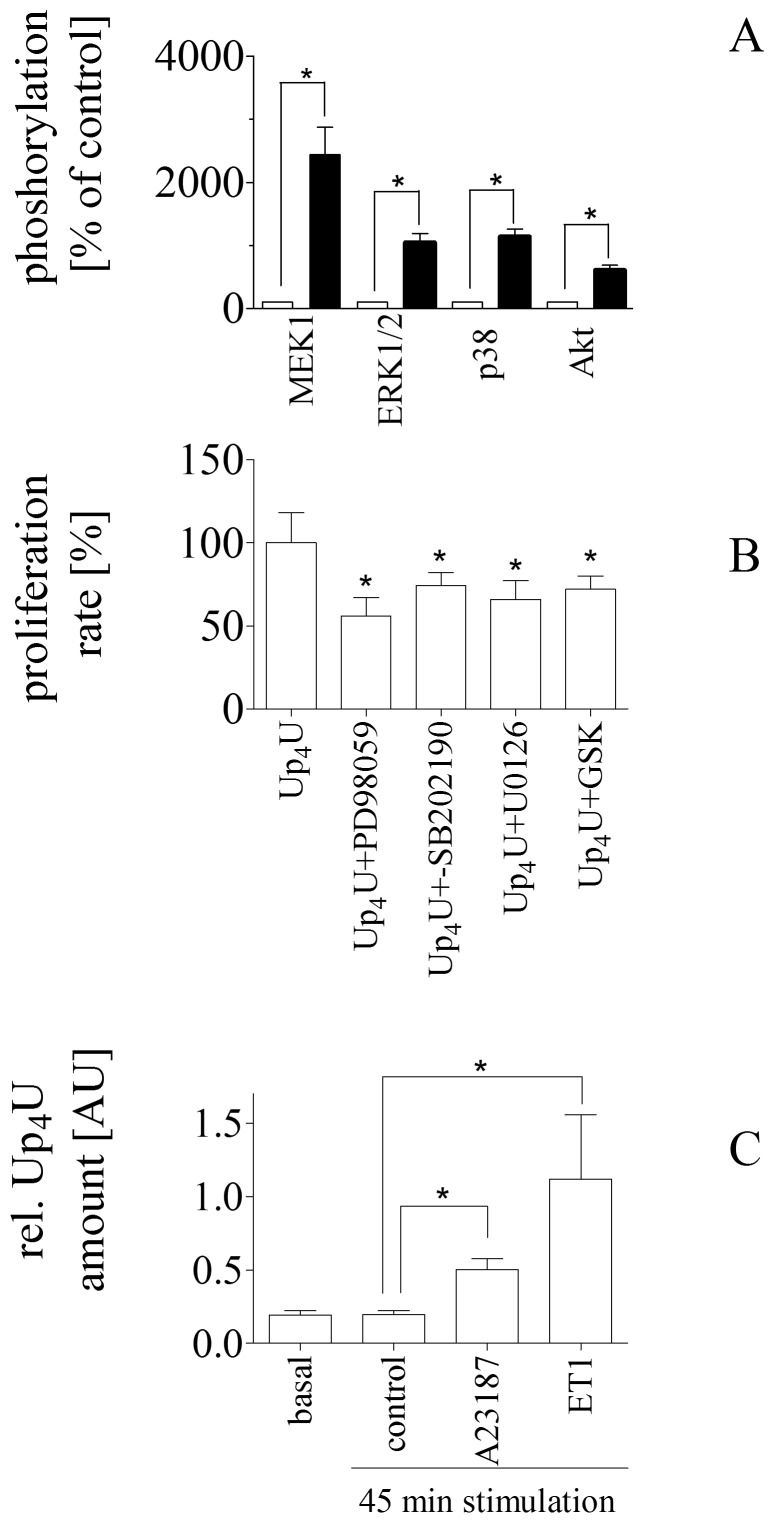
(**A**)Effect of Up_4_U on phosphorylation of MAPK. Phosphorylation of MEK1, ERK1/2, Akt, and p38 measured by Luminex™ technique before (open bar) and after stimulation with Up_4_U for 10 min (filled bar). Ratio of phospho/total were normalized to protein content of the lysates and demonstrated as percent stimulation relative to control (*p<0.05; n = 5). (**B**)Effect of PD98059, U0126, SB2021902, and GSK on Up_4_U induced proliferation rate in vascular smooth muscle cells (**C**)Up_4_U amount of secretome from in-vivo/ex-vivo stimulated aortic rings. (*p<0.05; n = 4).

We were interested to elucidate by which intracellular pathway Up_4_U can mediate proliferation. We tested potential activation of Mapkinases and can show that in a time-dependent way Up_4_U activates p38, MEK1, ERK1/2 and Akt with a maximal stimulation after 10 min (**Figure 4** **.A**). In the presence of U0126 (MEK1-inhibitor), SB202190 (p38 inhibitor), PD98059 (Erk1/2 inhibitor), and GSK690693 (Akt-inhibitor), Up_4_U induced proliferation was significantly reduced, indicating that all mapkinase activation is involved in the proliferative response (**Figure 4** **.B**).

Finally, the in-vivo/ex-vivo ability of endothelial cells to release Up_4_U was assessed in freshly isolated rat aortic rings (**Figure 4** **.C**). Up_4_U was detected in the 24 h conditioned media of freshly isolated aortic rings and in a subsequent 45 min. control conditioned media. Up_4_U content further increased following stimulation with either calcium ionophore (A23187) or endothelin 1.

## Discussion

Up_4_U is a potent angiogenic factor in human vascular endothelial cells. We tested the actions of Up_4_U on three major mechanisms contributing to angiogenesis, namely migration, proliferation, and tube formation [29]. Up_4_U directly increased the proliferation rate of endothelial cells, stimulated migration and tube formation, sprouting of endothelial cells and potentiated the proliferative effects of a peptidic growth factor, PDGF on vascular smooth muscle cell proliferation. Migration is only stimulated with higher Up_4_U concentrations, which are not present in the plasma of healthy humans, but may be reached locally upon release of Up_4_U into the extracellular space. In order to asses the in vivo relevance of these findings, Up_4_U was assayed in the supernatants of freshly isolated aortic rings. Aortic rings released Up_4_U into the supernatant under non-stimulated conditions and known inducers of Up_4_U release further increased the Up_4_U content in conditioned media.

Up_4_U belongs to the group of dinucleoside polyphosphates, which regulates vascular tone [15], vascular smooth muscle proliferation [30]–[32], platelet aggregation [33] and mesangial cell proliferation [34]. Up_4_U is the first member of family of a dinucleoside polyphosphates which has only pyrimidine-containing nucleosides at both ends. Dinucleoside polyphosphates were isolated from body fluids and cells like platelets [35], [36], brain [37], heart [38], plasma [39] or endothelial cells [15]. Dinucleoside polyphosphates are released into the circulation from several cell types, including activated platelets [36], [40], [41], chromaffin cells of the adrenal glands [37], [42]–[44], tubular cells [32], [45] or from synaptic vesicles [46]. Furthermore, dinucleoside polyphosphates are important neurotransmitter molecules in the nervous system [47]. Dinucleoside polyphosphates occur in human plasma at concentrations sufficient to cause vasoregulatory effects [39], which are significantly increased in pathophysiological conditions, like hypertension [48]. The very last one was Up_4_A which acts as potent endothelium derived contracting factor [15]. The actions of the currently known dinucleoside polyphosphates have been extensively reviewed recently [49].

Recently the vascular endothelial growth factor receptor 2 (VEGFR2) was described as capable of synthesizing the dinucleoside polyphosphates uridine adenosine tetraphosphate (Up_4_A), diadenosine polyphosphates (Ap_x_A; with x = 2–6), adenosine guanosine polyphosphates (Ap_x_G; with x = 2–6) as well as diguanosine polyphosphates (Gp_x_G; with x = 2–6) [50]. Therefore, it is likely that Up_4_U is synthesized by the VEGFR2 in-vivo, too.

Which receptors mediate the Up_4_U effects? Endothelial cells migration is significantly inhibited by suramin. From the P2Y receptors expressed in endothelial cells, suramin inhibits the P2Y1, P2Y2 and P2Y6, but not the P2Y4 subtype [51]. Suramin markedly inhibits migration in our experiments. On the other hand RB2, which is known to block both P2Y1 and P2Y6 receptors [51], did not show a significant effect. Therefore, the P2Y2 receptor appears to be the subtype involved in the stimulatory effects of Up_4_U on endothelial cells migration. The non-significant effect of PPADS, which is an inhibitor of P2Y1 receptors [51], is also compatible with this view. ATPγS is a selective activator of P2Y2 receptors and can mimic the effect of Up_4_U on proliferation. Up_4_U induced proliferation is intracellularly mediated by Mapkinase activation.

In contrast to VEGF and adenosine, which are produced by a multitude of tissues primarily as a response to hypoxia [52], [53], Up_4_U seems to be produced mainly by endothelial cells in an autocrine fashion. Moreover, the experiments using shear stress suggest that hemodynamic rather than metabolic factors regulate Up_4_U secretion. Thus with respect to production and regulation, Up_4_U differs from the most important known peptidic and non-peptidic angiogenic factors.

It would appear that Up_4_U acts synergistically with peptidic growth factors. Up_4_U stimulates vascular smooth muscle cell proliferation only when applied in combination with a peptidic growth factor like PDGF, but this costimulatory effect is even present with nanomolar concentrations, which are also found in human plasma.

To what extent are the angiogenic effects of Up_4_U different from those of the nucleotides known to affect angiogenesis like ATP and UTP? Our experiments show that both ATP and UTP do not stimulate VSMC cells growth, either alone or in combination with PDGF. Both ATP and UTP stimulate migration, but the effects are markedly less than that of Up_4_U. Therefore, although UTP could be generated as a split product of Up_4_U, it seems unlikely that Up_4_U exerts its effects by split products such as UTP. Additionally, Up_4_U effects sprouting effects of endothelial cells.

What is the role of Up_4_U-induced angiogenesis in the context of other angiogenic factors? Up_4_U is secreted from endothelial cells upon stimulation by shear stress. This mechanism of release suggests that Up_4_U may mediate angiogenic stimuli from vascular endothelial cells. On the other hand, adenosine is known to mediate hypoxia-induced angiogenesis [54]. Also VEGF is secreted mainly as a response to hypoxia [55]. Up_4_U may be regarded as a further angiogenic factor secreted upon other stimuli than the known angiogenic factors and possibly modulating their actions.

In summary Up_4_U is a novel human endothelium-derived angiogenic nucleotide, which acts on human endothelial cell migration, proliferation, tube formation and induce sprouting of endothelial cells. With respect to VSMC proliferation Up_4_U acts synergistically with peptidic growth factors. These autocrine angiogenic effects of Up_4_U are mainly regulated by stimulation of EC.

## Supporting Information

Figure S1 (A) Reversed phase chromatography of deproteinized supernatants from stimulated endothelial cells. The fraction for further fractionation is labelled by an arrow. (B) Affinity chromatography of the fraction labelled by an arrow in **Figure S1.A** by using a phenylboronate affinity column. The fraction for further fractionation is labelled by an arrow. (X) Reversed phase chromatography of the fraction labelled by an arrow in **Figure S1.B**. The fraction for mass-spectrometric analysis is labelled by an arrow. (Δ) Molecular structure of diuridine tetraphosphate.
(TIF)
