# Correction: Identification of a Potent Endothelium-Derived Angiogenic Factor

**DOI:** 10.1371/annotation/0c4c8143-c10e-4fe0-b944-b1fcd79995e7

**Published:** 2013-08-13

**Authors:** Vera Jankowski, Markus Tölle, Thi Nguyet Anh Tran, Markus van der Giet, Mirjam Schuchardt, Kerstin Lehmann, Doreen Janke, Burkhard Flick, Alberto Ortiz, Maria D Sanchez-Niño, Martin Tepel, Walter Zidek, Joachim Jankowski

The names of two authors were given incorrectly. The correct names of the ninth and tenth authors are, respectively: Alberto Ortiz and Maria D Sanchez-Niño. The correct abbreviation of the tenth author in Contributions is: MDSN 

